# The Use of Antimalarial Drugs against Viral Infection

**DOI:** 10.3390/microorganisms8010085

**Published:** 2020-01-08

**Authors:** Sarah D’Alessandro, Diletta Scaccabarozzi, Lucia Signorini, Federica Perego, Denise P. Ilboudo, Pasquale Ferrante, Serena Delbue

**Affiliations:** 1 Department of Biomedical, Surgical and Dental Sciences, University of Milano, 20133 Milan, Italy; sarah.dalessandro@unimi.it (S.D.); lucia.signorini@unimi.it (L.S.); federica.perego@unimi.it (F.P.); pasquale.ferrante@unimi.it (P.F.); 2 Department of Pharmacological and Biomolecular Sciences, University of Milano, 20133 Milan, Italy; diletta.scaccabarozzi@unimi.it; 3 Département des Sciences de la Vie, University of Fada N’Gourma (UFDG), Fada N’Gourma BP 54, Burkina Faso; denise.ilboudo@gmail.com

**Keywords:** antimalarial drugs, viruses, emerging infections

## Abstract

In recent decades, drugs used to treat malaria infection have been shown to be beneficial for many other diseases, including viral infections. In particular, they have received special attention due to the lack of effective antiviral drugs against new emerging viruses (i.e., HIV, dengue virus, chikungunya virus, Ebola virus, etc.) or against classic infections due to drug-resistant viral strains (i.e., human cytomegalovirus). Here, we reviewed the in vitro/in vivo and clinical studies conducted to evaluate the antiviral activities of four classes of antimalarial drugs: Artemisinin derivatives, aryl-aminoalcohols, aminoquinolines, and antimicrobial drugs.

## 1. Introduction

Antimalarial drugs used for the treatment and prevention of malaria are classified in a heterogenic group [1]. They are usually divided based on the chemical structure or the source of the drugs. Most of them derive from traditional medicine and plants. After identification of the active principles, chemical modifications are introduced to increase the activity and ameliorate the selectivity index. They present different modes and various mechanisms of action, which are often still not elucidated, against malaria parasites. Furthermore, due to the complexity of these molecules, additional side activities have been reported. For these reasons, antimalarial drugs have been studied, proposed, and sometimes used for the treatment of other pathologies, such as cancer, autoimmune diseases, and nonmalaria infectious diseases [2,3,4]. Moreover, the geographical overlaps between malaria and viral-related diseases [5,6,7] have led to the consideration of possible use of antimalarial drugs as new antiviral drugs. Finally, the lack of new effective antiviral drugs and vaccines against many viral infections has strengthened interest in the potential antiviral activity of antimalarial drugs.

**Figure 1:**
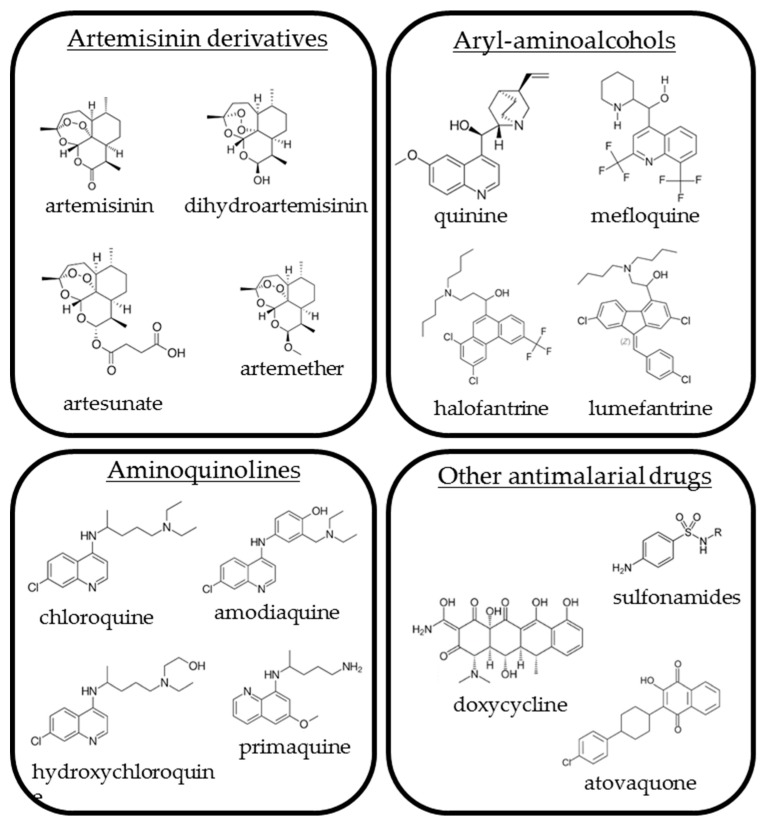
Chemical structures of the compounds described in the text.

In the present review, the authors present the use and the efficacy against human viruses of the principal antimalarial drugs, divided into four main groups: Artemisinin derivatives, aryl-aminoalcohols, aminoquinolines, and antimicrobial drugs. The chemical structures of the cited compounds are summarized in Figure 1. Works on newly synthesized derivatives, which are not licensed, were not taken into consideration. When possible, original manuscripts were cited. However, previous works of other scientists were acknowledged also by citing review articles, aiming to provide a comprehensive list of published papers on the proposed field.

## 2. Artemisinin Derivatives

*Artemisia annua* (qinghao) is a plant of the *Asteraceae* family, which has been used for ages in traditional Chinese medicine [8]. The sesquiterpene lactone artemisinin (ART), the active principle, was discovered in the 1970s. Since then, chemical structural modification studies have been performed to obtain new compounds with enhanced antimalarial activity and improved pharmacological properties. ART derivatives are safe and well-tolerated drugs. This safety is one of the reasons why they have been studied for their efficacy in other diseases beyond malaria. ART derivatives are active against other parasites, cancer cells and viruses, although with lower potency, with effective concentration_50s_ (EC_50_s) in the micromolar range, compared to the nanomolar range as antimalarials [9].

The majority of the literature describes the antiviral effect of ART derivatives in vitro toward human cytomegalovirus (HCMV). A large contribution to the field was given by Thomas Efferth and his collaborators, with both original research articles [10,11,12,13] and reviews [14,15].

### 2.1. Artemisinin

ART, the active principle extracted from *Artemisia annua*, is poorly soluble in water and oil, thus, the development of semisynthetic derivatives was necessary for appropriate formulation. Poor physicochemical properties may account for the scarce literature about the use of ART as an antiviral. ART, dihydroartemisinin (DHA) and artesunate (AS) were compared for their anti-HCMV effect in a fibroblast cell model by measuring viral DNA synthesis in cellular lysates. ART showed the lowest activity, even when fractional doses and daily repeated administration were used to counteract the problem of instability of the compounds in a culture medium [16]. Compared to other compounds from traditional Chinese medicine, ART and AS were also the most active, with low toxicity, for the inhibition of the hepatitis B virus (HBV), measured by hepatitis B surface antigen (HBsAg) and DNA release in a culture medium. Moreover, synergism with the antiviral lamivudine was demonstrated [17]. ART downregulated the oncogenic human papillomavirus (HPV) 39 proteins E6 and E7 in an in vitro model of cervical carcinoma [18]. These results partially confirmed the report by Disbrow and colleagues, who observed an antiproliferative effect of DHA on canine oral papillomavirus [19].

ART also inhibits hepatitis C virus (HCV) replicon replication [20], and the effect is synergistic with hemin, an iron donor [21,22].

Finally, ART showed inhibition of human immunodeficiency virus (HIV) replication, but the effect was not reproducible in different cell models [23].

### 2.2. Artesunate

#### 2.2.1. Artesunate and HCMV

The activity of AS on the replication of HCMV was demonstrated in different in vitro cell models, such as fibroblasts [11,16,24] and tumor cells [25,26]. Compared to other ART derivatives, AS had the highest activity [16,27] and an activity comparable to or even higher than that of classical antiviral drugs, such as ganciclovir [16,25,28]. The antiviral effect was confirmed against different, multidrug-resistant strains [10,25,27,29] and with different methods, such as the plaque assay and luminescent/fluorescent methods based on the use of transgenic viral strains [24,30,31]. In some cases, the effect of AS was synergistic with antiviral drugs such as maribavir [27,32,33], lamivudine [17], ganciclovir [34], foscarnet, cidofovir, letermovir [33]. However, in vitro data regarding synergism are not always confirmed in other experimental conditions, as for the case reported by Morère, where synergism between maribavir and AS was not confirmed in an ex vivo placenta model [32]. Moreover, few data obtained with animal models are available. In the rat CMV/rat model, AS demonstrated antiviral activity, measured as the dissemination of virus to the salivary glands, the number of viral genome copies, and virus titers in salivary glands. This activity was exerted only when AS was coadministered with iron in the Ferrosanol™ formulation [11]. AS in association with valacyclovir was tested in a murine model of herpes simplex virus encephalitis (HSE) to evaluate not only the antiviral but also the immunomodulating activity, which would be beneficial in this disease model. The survival rates of mice treated with both drugs were higher than those of mice treated with valacyclovir alone, but no significant difference was observed in the brain viral loads. Levels of cytokines such as Interleukine (IL)-1β, IL-2, IL-6, Interferon (IFN)-γ, and CCL2 were reduced in mice treated with valacyclovir combined with ART versus those in mice treated with valacyclovir alone [35].

Clinical data supporting the use of AS against HCMV are contrasting. In a case report, a rapid decrease in viral load was observed in a stem cell transplant recipient infected with a newly identified foscarnet-resistant and ganciclovir-resistant HCMV strain and treated with AS [12]. In this regard, a clinical trial was registered for the use of AS in stem cell transplant recipients, but although the recruitment of 20 patients in Israel has been completed, no results are available yet (Table 1).

On the other hand, a renal transplant recipient patient with documented valganciclovir resistance mutations in HCMV was treated with AS, with no positive effects [46]. A third case report of a patient with multidrug-resistant HCMV infection is difficult to interpret due to the complicated series of treatments after two hematopoietic stem cell transplantations and consequent acute graft-versus-host disease episodes. In this patient, AS given in association with maribavir was withdrawn two weeks after initiation because of orthostatic hypotension [47]. Wolf and colleagues described six cases of stem cell transplant recipients who received pre-emptive AS treatment for HCMV infection. Two of these showed a decrease in the viral load [13]. In another study, five transplanted patients infected with HCMV strains resistant to different antiviral drugs were treated with AS after many unsuccessful cycles of antiviral treatments. Three out of five had a favorable outcome [48].

At present, ART derivatives are recommended as antimalarial treatments in combination with another molecule with a different mechanism of action and longer half-life (ART combination therapy, ACT) to avoid the onset of resistance and to prevent recrudescence. One of these combinations, AS plus amodiaquine (AQ), was tested against HCMV in a clinical study conducted on 494 Ugandan children treated for acute malaria either with the ACT or with sulfadoxine–pyrimethamine plus AQ. No measurable difference was observed in either the HCMV detection frequency or load in the blood of children in the two groups [49].

#### 2.2.2. Artesunate and Other Viruses

AS was effective at a low micromolar range against Epstein Barr virus (EBV) in both epithelial cells and lymphocytes [50]. Antiviral activity against human herpes virus-6 (HHV-6) was demonstrated not only in vitro [51] but also in a child affected by HHV-6B-associated myocarditis. AS treatment was associated with a decrease in the levels of HHV-6B DNA in the myocardium [52]. However, data on the effect of AS against HHV-6 are contrasting in the literature [53].

AS affects human BK polyomavirus (BKPyV) and JC polyomavirus (JCPyV) replication in vitro [54,55]. Both viruses latently and asymptomatically infect the human host and are able to reactivate in immunosuppressed hosts, such as HIV-positive patients or transplant recipients. JCPyV causes a rare and fatal disease known as progressive multifocal leukoencephalopathy (PML), while BKPyV is associated with nephropathy [56].

As mentioned above, ART and AS were more active against HBV and less toxic than other compounds from traditional Chinese medicine [17]. The reduction in HCV replicons caused by AS was dose and time-dependent in vitro and increased when AS was given in association with IFN [57].

Interestingly, in the USA, an open-label study is currently investigating a novel nonsurgical approach to the treatment of HPV-associated anal intraepithelial high-grade neoplasia using AS suppositories. The outcomes that will be verified will be the regression of the lesions and the clearance of HPV. Similarly, a phase II double-blind, placebo-controlled, randomized study of AS vaginal inserts has been designed for the treatment of women who have cervical high-grade intraepithelial neoplasia, but recruitment has not yet started (Table 1).

Finally, the combination AS-AQ was also used in patients infected with the Ebola virus (EBOV), reducing the mortality risk more than the other ACT used (artemether-lumefantrine) [58]. However, moderate to serious risk of bias and small sample sizes preclude conclusions [59]. During the EBOV disease epidemic in West Africa in 2014–2016, two mass drug administrations of AS-AQ were implemented to decrease the burden of malaria. Garbern and colleagues performed a retrospective study to assess the potential effect of this treatment on the mortality of patients with EBOV. Although the risk of mortality for treated patients compared to that of EBOV infected patients not exposed to AS-AQ was decreased, the effect was not significant. Prospective trials are needed [60].

### 2.3. Other Artemisinin Derivatives

The activity of different ART derivatives was evaluated against HCMV in a fibroblast model using a luminescent assay based on transgenic viral strain. Compared to AS and/or ART, artemether was the most active. However, the activity was only seen at the micromolar range of concentrations, and only the synthesis of dimers allowed the activity to be effective at nanomolar concentrations [61,62].

DHA is the active metabolite of most ART derivatives and an antimalarial drug itself. DHA and ART were tested against bovine viral diarrhea virus (BVDV), a surrogate in vitro model of HCV, showing moderate activity in the micromolar concentration range [63].

Artemether is often used in combination with lumefantrine. One of the commercial versions of this combination, Coartem^®^, was used in a prospective observational study in Mali in children (6 months–10 years) coinfected with HCMV and malaria. Viral load in the urine decreased but only in high virus shedders [64]. The artemether-lumefantrine combination was also used in patients infected with EBOV, showing reduced efficacy in reducing the risk of death compared to that of AS-AQ, as already described in the “Artesunate” paragraph [58].

## 3. Aryl-Aminoalcohols

The antiviral effect of the aryl-aminoalcohol compounds quinine sulfate, mefloquine, halofantrine, and lumefantrine on both classical and emerging viruses has been studied.

### 3.1. Quinine Sulfate

Quinine, an alkaloid extract from *Chinchona* (quina-quina) tree bark, was discovered in the 17th century and has been used to treat malaria since the early 1600s, currently still playing a pivotal role, especially in the treatment of chloroquine (CQ)-resistant *Plasmodium falciparum* [65,66]. Due to the benefit derived from antimalarial drugs in other pathologies, possible antiviral effects of quinine were investigated.

The first manuscript regarding the effect of quinine on influenza virus infections in mice was published in 1946 [67].

Subsequently, in vitro evaluation of quinine sulfate has been conducted with other viruses, such as herpes simplex virus-1 (HSV-1) and influenza A virus (IAV). Quinine sulfate at micromolar but not toxic doses reduced the number of plaques formed by HSV-1 in vitro in Vero and HaCaT cell models, although no viricidal activity was observed [68,69]. Quinine sulfate in vitro activity was also tested against IAV by means of viral plaque inhibition assay, evaluating its prophylactic activity and showing different effects with an EC_50_ within the micromolar range, depending on the viral strains [70]. Recently, quinine sulfate was tested in vitro against emerging dengue virus (DENV) strains in different cell lines, showing a reduction in DENV-2 virion production up to 80% compared to that of the untreated control and a concentration-dependent reduction in DENV RNA and viral proteins. The inhibition of replication was then confirmed for all four different serotypes of DENV [66].

### 3.2. Mefloquine

#### 3.2.1. Mefloquine and JCPyV

Mefloquine (MQ), a synthetic analog of quinine with a long history of use and good safety in humans, has been widely tested as an antiviral. One of the first reports refers to JCPyV. In 2009, Brickelmaier et al. chose MQ because of its high blood-brain barrier penetration capability since it accumulates in brain tissue at a six-fold higher concentration than its EC_50_. MQ was active against different strains of JCPyV in three different cell models, with EC_50_s within the low micromolar range [71]. Because of the absence of a suitable animal model, this first paper represented the starting point for a series of trials in human populations, which obtained contrasting results and are summarized in Table 2. Seventeen case reports described the use of MQ at different doses [72,73,74,75,76,77,78,79,80,81,82,83,84,85,86,87,88]. Although in 12 cases there was no progression of the disease at follow-up [72,73,74,77,78,79,81,82,83,84,86,87], it is difficult to draw conclusions due to the challenging treatment protocols and compromised health of the patients. In many cases, MQ was combined with mirtazapine, an antidepressant that, acting on the 5-HT2A serotonin receptor, is able to inhibit JCPyV entry into glial cells, preventing the diffusion of the infection in oligodendrocytes. The outcomes of this treatment are controversial, leading to the resolution of the infection, with a claimed effect of MQ and mirtazapine treatment [86,87,89,90,91,92,93,94,95,96,97,98,99,100,101,102,103,104,105], and to the resolution of the infection probably due to other factors [96,106,107,108,109] or to the death of the patient, which was not always directly related to the unsuccessful therapy [87,96,110,111,112,113,114,115,116]. In one case, the suspension of the therapy was necessary due to the side effects [117]. In a few cases, a third partner drug was added to MQ and mirtazapine. Again, the outcome was variable, and the contribution of the single drugs was difficult to determine [118,119].

MQ has also been combined with risperidone [75] or risperidone and cytarabine [120], and the final outcomes were opposing, either death or recovery of the patient.

In one case, MQ was administered with the antiviral cidofovir, resulting in final remission of the pathology, most likely due to synergy with other factors [121].

Different case reports have described the use of MQ by itself in HIV-positive PML patients, sometimes with repression of JCPyV replication [122,123,124,125] and rarely with the death of the patient [126]. The combined treatment MQ and mirtazapine was administered to HIV-positive patients, leading to a failure because of premature death and not always due to the PML itself [127,128].

One randomized study was conducted with HIV-positive and HIV-negative patients, comparing the efficacy of the standard of care normally used to treat PML to the standard of care supplemented with MQ. This study ended prematurely because of a lack of significant differences between the two groups [129].

#### 3.2.2. Mefloquine and Other Viruses

A study on the antiviral effect of MQ on IAV was performed by Marois et al. MQ showed different grades of efficacy, depending on the viral strain and ranging from partial inhibition of replication to total ineffectiveness [70].

More recently, the effect of MQ on some emerging viruses was studied. MQ was tested for the first time against the Zika virus (ZIKV) in 2016, showing different reductions in infection rate, depending on the cell model used, and different cytotoxicities, thus making it difficult to draw a conclusion [130]. Balasubramanian et al. confirmed the in vitro effect of MQ on ZIKV infection and evaluated it on DENV, performing several in vitro assays [131]. Sun et al. performed an in vitro screening of 795 fixed-dose drug combinations of three molecules, choosing those able to block more than 90% EBOV-like particle entry into HeLa cells. One of the three best combinations was composed of MQ with toremifene (an antagonist of estrogen receptors) and the antifungal posaconazole, whose activity was confirmed by dose-response experiments in Vero cells infected with EBOV [132].

Although some benefits could be seen in the use of MQ as antiviral drug, its neurotoxicity should be taken into account: when it is used for malaria prophylaxis, it is known to cause serious neuropsychiatric adverse reactions, and to date, international MQ labels warn patients to discontinue it at the onset of prodromal psychiatric and neurologic symptoms [133].

However, to date, to the best of our knowledge, there are no systematic studies concerning neurotoxic manifestations of MQ, when used ad antiviral drug, and not for the antimalarial prophylaxis. It could be speculated that in some conditions the benefit:risk ratio would look more favorable than for MQ used for malaria chemoprophylaxis.

### 3.3. Halofantrine and Lumefantrine

While good results were obtained using quinine sulfate and MQ as antivirals, the same cannot be stated for halofantrine and lumefantrine, which failed in the inhibition of viral replication, based on the few studies conducted to date. Mazzon and colleagues, performing an in vitro screen of ~2500 compounds, were able to describe inhibition activity of halofantrine on Semliki Forest virus (SFV) and DENV-2, but, due to the low selectivity index of the drug, further investigations were not conducted [134].

Lumefantrine has been tested only in a commercial combination with artemether, known as CoArtem^®^, as previously described in the paragraph about ART derivatives.

## 4. Aminoquinolines

### 4.1. Chloroquine and Hydroxychloroquine and Emerging Viruses

Chloroquine (CQ) is an aminoquinoline known since 1934. It was synthesized to be used as an antimalarial drug, but its properties and mechanism of action encouraged its use for the treatment of different diseases. Currently, CQ and its hydroxy-analog hydroxychloroquine (hydroxyCQ) cannot be used as antimalarial drugs in wide areas where the resistance of malaria parasites emerged. They are commonly used for connective tissue disorders, such as rheumatoid arthritis. Due to low toxicity and cost, high tolerability and immunomodulatory properties, CQ and hydroxyCQ have also been proposed for use against viral infections. Even if their specific mechanisms in individual diseases are not clear, it is well assessed that the antiviral activities of the aminoquinoline take advantage of their strong anti-inflammatory activity. The major proposed mechanisms of actions of CQ analogs which are suggested to influence the anti-viral activity are, among the others: the inhibition of cytokine production and release by T cells: IL-1, 2, 6, or 18, tumor necrosis factor TNF-α and IFN-γ, reduced levels of chemokines CCL2 and CXCL10, inhibition of micro-RNA expression, decreased TH17-related cytokines, decreased DNA, RNA and protein synthesis in thymocytes (reviewed in [135]).

The in vitro antiviral effect of CQ was first reported approximately 40 years ago [136,137], and since that time, its use as an antiviral drug has been extensively discussed. In particular, CQ/hydroxyCQ have been used for the treatment of emerging chikungunya virus (CHIKV) infection, recently causing numerous outbreaks in the world. Khan et al. showed that the treatment of infected Vero cells with different micromolar concentrations of CQ reduced virus yield and viral RNA copy number [138]. De Lamballerie and colleagues confirmed the inhibition of CHIKV replication in Vero-E6 cells using CQ. The efficacy of CQ was inversely related to the concentration of the viral inoculum used, an unfavorable observation, considering the high viremia measured at the acute stage of CHIKV infection (up to 10^10^ virus copies/mL serum) [37]. Sourisseau and colleagues treated HeLa cells with CQ, obtaining a potent inhibition of CHIKV replication and its relative cytopathic effects [139].

A double-blind placebo-controlled trial was designed to evaluate the efficacy and safety of CQ for the treatment of CHIKV infection in 2006 in French Reunion Island (Indian Ocean). No significant difference was observed between the CQ and placebo groups, either in the mean duration of febrile arthralgia or in the rate of viremia decrease [37]. However, the number of patients included in the study was too small to draw definitive conclusions regarding the efficacy of CQ treatment (Table 1) [37]. Aminoquinolines were proposed for the treatment of other viral infections, such as ZIKV. In 2017, it was demonstrated that CQ and AQ exerted anti-ZIKV activity in Vero cells, with low micromolar IC_50_s [140]. These results were in agreement with the decreasing number of ZIKV-infected cells after CQ treatment. Additionally, CQ protected the cells from further ZIKV infection, as measured by cell viability at noncytotoxic concentrations [141].

The activity of CQ has also been indirectly demonstrated against DENV infection. The results obtained by Kleber and colleagues showed that CQ suppressed TNF-α and IFN-γ production, and it was hypothesized that CQ might be used to treat patients suspected of having dengue disease, avoiding the more severe form of dengue hemorrhagic fever and/or shock. A clinical trial was also established to verify the effect of CQ versus placebo in DENV-infected patients in Brazil. CQ promoted a reduction in the intensity of pain and an improvement in the well-being of patients with DENV infection but did not alter the duration of the disease or the intensity and days of fever (Table 1) [36].

To study the effects of CQ against EBOV, a group led by Dowall conducted an in vitro investigation using the human cell line MRC-5 and in vivo studies with the well-characterized guinea pig model [142]. They were able to demonstrate that CQ reduced EBOV replication in MRC-5 cells. In contrast, the administration of CQ to 12 Guinea pigs did not protect the infected animals against the Ebola disease [142]. Madrid and colleagues suggested that CQ could interfere with the late stages of EBOV replication and assembly [143]. Despite these positive in vitro results, the clinical trials were sometimes conflicting. For this reason, later, the literature was reviewed to clarify the efficacy of CQ in the treatment of filovirus infection [144]. It was concluded that the efficacy of CQ against the viruses belonging to this family was dependent on the CQ plasma concentrations, which must be sustained in patients until the clearance of the viremia [144].

CQ was shown to inhibit the replication and spread of coronavirus (CoV) in vitro and to prevent infection with CoV in newborn mice. Since the suppressive effect of CQ was also present when the cells were treated before the infection, a prophylactic advantage of CQ use was suggested [145,146,147].

### 4.2. Chloroquine and Hydroxychloroquine and HCV

CQ and its analogs have effects against HCV. In particular, the treatment of JFH-1 or Huh-7 cells with CQ reduced HCV entry, replication, and infection in a dose-dependent manner [148,149,150]. Furthermore, CQ, in combination with IFN-α, prevented the replication of HCV and enhanced the antiviral effect of IFN-α [149].

In this regard, two phase I/II clinical trials were initiated to verify the efficacy of the combination treatment of hydroxyCQ and ribavirin, but no results were posted due to limited recruitment.

### 4.3. Chloroquine and Hydroxychloroquine and HIV

The anti-HIV-1 and anti-HIV-2 activities of CQ and its analogs were tested in vitro and in vivo. The first report about the in vitro use of CQ as an anti-HIV-1 agent was published in 1990 by Tsai et al., which showed the suppressive effects of CQ on the replication of HIV-1 in a T cell line [151]. A few years later, Sperber and colleagues confirmed these results, showing the ability of CQ and hydroxyCQ to inhibit HIV-1 replication not only in T cells but also in monocytes [152,153]. Subsequently, the same group demonstrated the CQ and hydroxyCQ anti-HIV-1 and anti-HIV-2 in vitro effects at concentrations that are clinically achievable [154]. CQ had an additive effect against HIV-1 when used in combination with other antiretroviral agents [155,156]. Naarding et al. demonstrated that CQ reduced HIV-1 transmission to and replication in CD4 + T-lymphocytes [157]. Similarly, Martinson et al. observed that CQ had a preventive role in HIV infection, reducing CD8 + T cell activation upon HIV replication [158].

The antiviral activity of hydroxyCQ was demonstrated in vivo by several clinical trials. The somministration of hydroxyCQ was able to reduce the amounts of plasma HIV-1 RNA and IL-6 in patients treated for eight weeks compared to those of the placebo group [39]. During a second clinical trial, hydroxyCQ was shown to reduce the HIV-1 RNA plasma level, although at a lower level than the antiviral zidovudine [40]. In contrast, the results published in 2012 by Paton and colleagues showed negative results, with an increase in viral load and a decrease in CD4 number [41].

Another double-blind, randomized placebo-controlled trial testing the effects of CQ in 13 chronically HIV-infected persons was conducted in Minnesota. The results showed that the administration of CQ during chronic HIV infection resulted in decreased immune activation, but no data regarding HIV status were reported [43].

Very recently, the AIDS Clinical Trials Group A5258 was completed. It was a randomized, double-blind, placebo-controlled study in 33 HIV-1-infected participants off antiretroviral therapy and 37 participants on antiretroviral therapy. CQ modestly reduced immune activation in antiretroviral therapy-treated HIV-infected participants [42].

Finally, the recruitment of 1499 patients was concluded a few months ago in a randomized, controlled, open-label, phase III trial of the standard of care with CQ prophylaxis compared to no prophylaxis in HIV-positive patients in Malawi (Table 1) [44,45].

#### Chloroquine and Hydroxychloroquine and Other RNA Viruses

CQ was shown to inhibit the in vitro replication of H1N1 and H3N2 IAV strains [159]. A phase II clinical trial aiming to verify the effect of CQ compared to that of placebo on IAV was started in Singapore in 2005, and 1516 patients were recruited. However, CQ was not shown to prevent infection with IAV (Table 1) [38].

CQ had inhibitory effects on the entry and replication of enterovirus (EV)-A71 in cell-based systems [160]. Yong and colleagues studied the efficacy of CQ against several EV serotypes and evaluated its therapeutic capacity in vitro in RD cells and in vivo in a murine model [161]. They demonstrated the potential of CQ as an antiviral in the treatment of hand, foot, and mouth disease caused by EV infection. The positive results obtained in the murine model of infection were indicative of the fact that CQ may mitigate the disease severity in mammals [161].

### 4.4. Amodiaquine and Emerging Viruses

Amodiaquine (AQ) was originally developed and has been widely used for the treatment of malaria. However, subsequent studies revealed that it was active against a wide range of human pathogens, including several viruses. In a study published in 2014, it was investigated whether quinolone derivatives could inhibit the replication of DENV [162]. The time-course analysis suggested that AQ was stable and that it reproducibly inhibited DENV infectivity. The data also showed that viral entry and internalization were partially inhibited by the drug, but the major effect occurred at a later stage of the viral life cycle [162].

It is known that AQ inhibits EBOV replication in vivo [58]. A recent study demonstrated that AQ was active against severe fever with thrombocytopenia syndrome (SFTS) caused by SFTS virus (SFTSV) [163].

### 4.5. Primaquine

Primaquine was tested as an antiviral on primary chicken embryo cells (CECs) infected by Newcastle disease virus [164]. It was demonstrated that primaquine had an effect on the accumulation of viral hemagglutinin on the cell surface. In addition, primaquine inhibited protein synthesis in virus-infected cells [164].

## 5. Other Antimalarial Drugs

### 5.1. Atovaquone

Atovaquone, a naphthoquinone antimalarial drug often used in pregnant women, is a ubiquinone (coenzyme Q) analog that inhibits mitochondrial cytochrome complex III (bc1 complex). It is also able to deplete the intracellular nucleotide pools by inhibiting dihydroorotate dehydrogenase, an enzyme for de novo pyrimidine synthesis [165]. In 2019, one published work reported the in vitro antiviral action of atovaquone against CHIKV. Low micromolar doses inhibited the number of infected cells, as evaluated by high-content fluorescence microscopy. The result was then confirmed by the reduction in CHIKV virions in different cells by plaque assay [164]. In the same paper, Kottkamp and colleagues evaluated the ability of atovaquone to reduce the infectivity of both a Brazilian and a Ugandan strain of ZIKV by immunostaining the envelope after treatment and infection and subsequent plaque assay in different cells. These data were further confirmed ex vivo in a human placenta tissue model, with a dose-dependent reduction in infection and virion production by the Ugandan strain [166].

### 5.2. Antimicrobial Drugs

Antibiotic drugs, such as doxycycline and sulfonamides, are widely used in the chemoprophylaxis of malaria in all malaria areas [167]. It has been reported that these therapies could have activities beyond antimalarial activity, also against viral infectious agents. Surprisingly, in many cases, the administration of antibiotics alone or in combination with other antiviral agents showed significant antiviral activities against different types of viral infections [168,169,170,171]. Despite this evidence, the antiviral mechanisms of action have not been completely investigated.

#### 5.2.1. Doxycycline

Doxycycline (DOX) is a semisynthetic tetracycline antibiotic that prevents bacterial protein synthesis by acting on ribosomes (30S subunit) [167,172,173,174,175]. DOX, widely used alone or in combination with quinine for chemoprophylaxis in CQ-resistant *P. falciparum* cases of malaria [176], has been shown to exert inhibitory effects against different microbial infections.

DOX significantly inhibited the proliferation of a panel of HPV-positive cervical cancer cell lines, also inducing apoptosis of cervical cancer cells in a time- and dose-dependent manner [177].

Concerning emerging infections, in a recent work, researchers tried to identify candidate antibiotics that can block the function of DENV viral envelope proteins to prevent viral entry. Using an innovative visual screening approach coupling computational studies and biologic assays on ten nontoxic candidates, they suggested that DOX significantly inhibited plaque formation, demonstrating an inhibitory effect on DENV propagation [178]. This result was confirmed by Rothan and colleagues, who evaluated the replication rate of DENV in an in vitro model of infected cells. They reported that DOX inhibited DENV replication in vitro by reducing viral protease activity and entry into host cells [168]. The same research group determined the inhibitory effects of DOX against CHIKV as well as its possible effect on the virus life cycle in Vero cells when the virus and the drug were administered concurrently to the cells. Computational studies indicated that DOX might be a noncompetitive inhibitor of CHIKV protease. This hypothesis was confirmed by in vitro experiments demonstrating that the effect of DOX was directed more toward viral entry than toward viral replication [169]. Furthermore, the inhibitory effect of DOX on the replication of vesicular stomatitis virus (VSV) in different stably infected cell lines was reported [170]. The results showed significant inhibition of the replication of VSV in a dose-dependent but not cell-type dependent manner, suggesting that DOX exerted its antiviral activity at the early-mid stage of VSV infection [170]. These results indicated a different mode of action of DOX against VSV than that against DENV and CHIKV, suggesting that the antiviral activity of DOX could be dependent on the virus species. Finally, only an in vivo study was published on IAV. Treatment with DOX attenuated acute lung injury in mice infected with a virulent IAV H3N2 strain, with no effects on virus titers, suggesting that the antibiotic treatment was able to alleviate severe influenza pneumonia symptoms [171]. It is, therefore, possible to conclude that despite the role of DOX as a potential antiviral agent in vitro, the mechanisms of viral replication inhibition and the targeted virus species have yet to be clarified. Clinical trials confirming in vitro observations are needed.

#### 5.2.2. Sulfonamides

Sulfonamides constitute an important class of drugs containing many types of pharmacological agents with broad-spectrum bacteriostatic activity. Sulfonamides interrupt the synthesis of folic acid, interfering with the dihydropteroate synthase and dihydrofolate reductase enzymes of bacteria (and protozoa) and inhibiting bacterial growth [179,180]. The sulfadoxine-pyrimethamine combination is used in some settings for the treatment of uncomplicated malaria in pregnant women, and it is the only drug currently recommended for intermittent preventive therapy during pregnancy [181]. A study investigated the effect of this combination on HIV replication. Experiments were conducted in vitro using peripheral blood mononuclear cells, MT-2 cells, MT-4 cells, and a latently infected cell line named U1. The results showed that the sulfadoxine-pyrimethamine combination combined with antiretroviral therapy significantly enhanced HIV replication in MT-2 cells, while it inhibited HIV replication in peripheral blood mononuclear cells [23]. Moreover, recent studies have demonstrated that sulfonamides can act on latent herpesviruses, such as EBV and Kaposi sarcoma herpesvirus (KSHV) [182,183]. Furthermore, a work conducted by Angius and colleagues reported that sulfonamide antibiotics suppressed the KSHV latent state in permanently infected lymphoma cells [183]. This result suggested that sulfonamides might play a potential role in clearing KSHV-infected lymphoma cells. Since conventional antiherpes drugs are able to slightly suppress viral replication in the lytic phase but do not clear the latent state, the finding of potential new small molecules, such as sulfonamides drugs, could initiate a promising program of studies on both oncogenic and degenerative diseases, in which herpesvirus latency is suspected to be involved [184,185,186].

## 6. Conclusions

Antimalarial drugs have been widely tested against a large number of viruses, especially in vitro, with variable outcomes (Table 3). Among the ART derivates, some showed strong activities against viruses, such as AS against HCMV, ART, and AS against HBV and HCV, and AS against HPV and HPyV, whereas data regarding the activity against HIV are uncertain.

Among the aryl-aminoalcohols, the use of MQ in the treatment of JCPyV infection has been extensive, although with contradictory outcomes. Among the aminoquinolines, both CQ and hydroxyCQ showed promising results in reducing the replication of some emerging viruses, such as DENV and ZIKV.

Emerging infections have also been targeted by antibacterial drugs, such as DOX. However, the successful use of antimalarial drugs in vitro did not always lead to a satisfactory outcome in their clinical application (Table 4).

Nevertheless, some drugs have already been used in several clinical trials, as summarized in Table 1. Most of them regard the use of antimalarial drugs against HIV infection, but some of them failed, and for others, the final results are not available. Although these outcomes can seem discouraging, at least four clinical trials deserve attention: The one on the use of AS against HCMV, with particular regard to the drug-resistant strains, the one targeting CHIKV with CQ, and the other two very innovative and ongoing trials on the use of AS against HPV for the treatment of anal and cervical intraepithelial high-grade neoplasia.

Based on these observations, we can state that the use of antimalarial drugs might be useful, especially in cases of antiviral resistance and in light of the emergence of many viruses against which effective drugs are not available.

## Figures and Tables

**Table 1:** Clinical trials for the evaluation of the activity of antimalarials on viral infections in patients.

Virus Family	Virus Species	Drug	Trial Number/Reference	Outcome
*Herpesviridae*	HCMV	Artesunate	NCT00284687	No results
*Papillomaviridae*	HPV	Artesunate	NCT03100045	Ongoing
			NCT04098744	Ongoing
*Flaviviridae*	HCV	Hydroxychloroquine	NCT01833845	Terminated due to failure to recruit subjects
			NCT01272310	Unknown
	DENV	Chloroquine	NCT00849602	Reduction in pain but not in length of disease [36]
	CHIKV	Chloroquine	NCT003913131	No difference between CQ- and placebo-treated groups [37]
*Orthomyxoviridae*	IAV	Chloroquine	NCT01078779	No prevention of IAV infection [38]
*Retroviridae*	HIV	Hydroxychloroquine	[39,40]	Reduction in HIV-1 RNA load in plasma
		Hydroxychloroquine	ISRCTN30019040	Increased HIV-1 replication and decreased CD4 numbers [41]
		Chloroquine	NCT00819390	Modest reduction in immune activation [42]
		Chloroquine	NCT02004314	Patients did not experience any improvement after CQ treatment [43]
		Chloroquine	NCT01650558	Terminated, awaiting results [44,45]

**Table 2:** Clinical studies assessing the efficacy of Mefloquine (MQ) treatment and its combinations in JCPyV-infected progressive multifocal leukoencephalopathy (PML) patients.

Case Reports			
HIV Status	Treatment	Outcome	References
HIV negative	Mefloquine	No PML progression	[72,73,74,77,78,79,81,82,83,84,86,87]
		Fatal outcome	[75,76,80,85,88]
	Mefloquine + mirtazapine	PML resolution with claimed effects	[86,87,89,90,91,92,93,94,95,96,97,98,99,100,101,102,103,104,105]
		PML resolution due to other factors	[96,106,107,108,109]
		Fatal outcome	[87,96,110,111,112,113,114,115,116]
		Therapy suspension due to side effects	[117]
	Mefloquine + mirtazapine + third partner drug	PML resolution	[118]
		Fatal outcome	[119]
	Mefloquine + risperidone	Fatal outcome	[75]
	Mefloquine + risperidone + cytarabine	PML resolution	[120]
	Mefloquine + cidofovir	PML resolution due to other factors	[121]
HIV positive	Mefloquine	No PML progression	[122,123,124,125]
		Fatal outcome	[126]
	Mefloquine + mirtazapine	Premature fatal outcome	[127,128]
**Clinical Trial**			
HIV negative versus HIV positive patients	Standard of care (SOC) versus SOC + mefloquine	Lack of differences: study ended prematurely	[129]

**Table 3:** In vitro/in vivo studies for the assessment of antimalarial drug activity against viral infection.

Virus Family	Virus Species	Drug	Type of Study (Model)	References
*Herpesviridae*	HSV-1	Quinine sulfate	in vivo	[68,69]
	EBV	Artesunate	in vitro	[50]
		Sulfonamides	in vitro	[182]
	HCMV	Artemisinin	in vitro	[16]
		Artesunate	in vitro	[10,11,16,17,24,25,26,27,28,29,30,31,32,33,34]
		Arthemeter	in vitro	[61,62]
	HHV-6	Artesunate	in vitro	[51,53]
	HHV-6B	Artesunate	in vitro	[52]
	KSHV	Doxycycline	in vitro	[183]
	HSE	Artemisinin	in vivo (mouse)	[35]
		Dihydroartemisinin	in vitro	[16]
	KSHV	Sulfonamides	in vitro	[183]
	RCMV	Artesunate	in vivo (rat)	[11]
*Polyomaviridae*	BKPyV	Artesunate	in vitro	[55]
	JCPyV	Artesunate	in vitro	[54]
		Mefloquine	in vitro	[71]
*Hepadnaviridae*	HBV	Artemisinin, artesunate	in vitro	[17]
*Papillomaviridae*	HPV	Artemisinin	in vitro	[18]
		Dihydroartemisinin	in vivo (dog)	[19]
		Doxycycline	in vitro	[177]
*Flaviviridae*	BVDV(surrogate HCV)	Artemisinin	in vitro	[187,188]
		Dihydroartemisinin	in vitro	[63]
	HCV	Artemisinin	in vitro	[20,21,22]
		Artesunate	in vitro	[57]
		Chloroquine	in vitro	[148,149,150]
	ZIKV	Mefloquine	in vitro	[130,131]
		Chloroquine	in vitro	[140,141]
		Amodiaquine	in vitro	[140]
		Atovaquone	in vitro	[166]
	DENV	Quinine sulfate	in vitro	[66]
		Mefloquine	in vitro	[131]
		Halofantrine	in vitro	[134]
		Doxycycline	in vitro	[168,178]
		Amodiaquine	in vitro	[162]
	CHIKV	Doxycycline	in vitro	[169]
		Chloroquine	in vitro	[37,138,139]
		Atovaquone	in vitro	[166]
*Togaviridae*	SFV	Halofantrine	in vitro	[134]
*Rhabdoviridae*	VSV	Doxycycline	in vitro	[170]
*Orthomyxoviridae*	IAV	Quinine sulfate	in vitro	[70]
			In vivo (mouse)	[67]
		Mefloquine	in vitro	[70]
		Doxycycline	in vivo (mouse)	[171]
		Chloroquine	in vitro	[159]
*Coronaviridae*	CoV	Chloroquine	in vitro	[145,146,147]
*Picornaviridae*	Enteroviruses	Chloroquine	in vitro	[160]
		Chloroquine	in vitro/in vivo (mouse)	[161]
*Filoviridae*	EBOV	Chloroquine	in vitro	[142,143]
			in vivo	[142]
		Artesunate, amodiaquine	in vivo	[58]
		Mefloquine	in vitro	[132]
*Retroviridae*	HIV	Artemisinin	in vitro	[23]
		Doxycycline	in vitro	[23]
		Mefloquine, toremifene, posaconazole	in vitro	[132]
		Chloroquine	in vitro	[151,157,158]
			in vivo	[155]
		Chloroquine, hydroxychloroquine	in vitro	[152,153,154,156]
*Phenuiviridae*	SFTSV	Amodiaquine	In vitro	[163]

**Table 4:** Clinical studies for the assessment of antimalarial drug activity against viral infection.

Virus Family	Virus Species	Drug	References
*Herpesviridae*	HCMV	Artesunate	[12,13,46,47,48]
		Artesunate-amodiaquine	[49]
		Arthemeter-lumefantrine	[64]
	HHV6	Artesunate	[52]
*Polyomaviridae*	JCPyV	Artesunate	[54,55]
		Mefloquine	[72,73,74,75,76,77,78,79,80,81,82,83,84,85,86,87,88,122,123,124,125,126]
		Mefloquine and mirtazapine	[86,87,89,90,91,92,93,94,95,96,97,98,99,100,101,102,103,104,105,106,107,108,109,110,111,112,113,114,115,116,117,127,128]
		Mefloquine, mirtazapine and a third drug	[118,119]
		Mefloquine and risperidone	[75]
		Mefloquine, risperidone and cytarabine	[120]
		Mefloquine and cidofovir	[121]
		Mefloquine and PML standard of care	[129]
*Filoviridae*	EBOV	Artesunate-amodiaquine	[58,60]

## Abbreviations

AQ	Amodiaquine
ART	Artemisinin
AS	Artesunate
CHIKV	Chikungunya virus
CQ	Chloroquine
DENV	Dengue virus
DHA	Dihydroartemisinin
EC_50_s	Effective concentrations
EBV	Epstein-Barr virus
HBV	Hepatitis B virus
HCMV	Human cytomegalovirus
HCV	Hepatitis C virus
HPV	Human papillomavirus
hydroxyCQ	hydroxychloroquine
IL	Interleukin
INF	Interferon
KSHV	Kaposi sarcoma herpesvirus
MQ	Mefloquine
RCMV	Rat cytomegalovirus
TNF	Tumor necrosis factor
VSV	Vesicular stomatitis virus
ZIKV	Zika virus
